# Advances in cancer immunotherapy for gastroenterological malignancy

**DOI:** 10.1002/ags3.12184

**Published:** 2018-06-29

**Authors:** Koji Kono

**Affiliations:** ^1^ Department of Gastrointestinal Tract Surgery Fukushima Medical University Fukushima Japan

Recent breakthrough results from immune checkpoint inhibitors (ICI) such as an anti‐cytotoxic T‐lymphocyte‐associated protein 4 (anti‐CTLA4) monoclonal antibody (mAb) (ipilimumab) and anti‐programmed cell death‐1 (anti‐PD‐1) mAbs (nivolumab and pembrolizumab) have paved the way to a new era of cancer immunotherapy, leading to a paradigm shift in cancer treatment. Currently, ICI has had immense clinical success resulting in sustained treatment response for a subset of cancer patients such as melanoma, non‐small‐cell lung cancer, urothelial carcinoma, squamous cell carcinoma of the head and neck, renal cell cancer, gastric cancer, and hepatocellular cancer.^1^, ^2^ Drug approval authorities such as the Food and Drug Administration (FDA) (USA) and the Pharmaceuticals and Medical Devices Agency (PMDA) (Japan) have approved clinical application for cancer treatment. Although ICI has made substantial progress, there are several problems to be improved from a clinical and translational aspect. The next two challenging steps for developing ICI are as follows. First is the identification of potential biomarkers that can predict the response to ICI. For example, in the case of anti‐PD1 mAb therapy, load of tumor‐mutation burden, expression of PD‐L1 on tumor cells, or degree of tumor‐infiltrating CD8 (+) T cells were reported to be potential biomarkers to predict responsiveness to anti‐PD‐1 mAb. The second challenge is the clinical development of combinatorial approaches. Interestingly, it was reported that combination of anti‐CTLA4 mAb with anti‐PD‐1 mAb led to “deep and rapid tumor regression” in almost one‐third of melanoma patients.^3^ With both anti‐CTLA4 and anti‐PD‐1 mAbs, some patients kept responding even after the treatment had been discontinued, suggesting their immune memory had been established.

Importantly, there has been limited success in the use of immunotherapy in the treatment of pancreatic cancer. In this issue of the *Annals of Gastroenterological Surgery*, Torphy et al comprehensively summarize the current status of immunotherapy for pancreatic cancer. They describe details of the complex tumor microenvironment of pancreatic cancer that is composed of immune cells, stromal cells, and extracellular matrix proteins. These complexes function within the microenvironment as important barriers to the effectiveness of cancer immunotherapy. For example, the tumor microenvironment is dominated by immunosuppressive cell types, including tumor‐associated macrophages (M2‐type macrophages), myeloid‐derived suppressor cells (MDSC), and regulatory T cells. Moreover, these immunosuppressive cells are regulated by carcinoma‐associated fibroblasts expressed by fibroblast activation protein (FAP)‐positive cells. Finally, the authors addressed that, in order to achieve efficient clinical response with ICI for pancreatic cancer, one should use a novel approach such as colony‐stimulating factor 1 receptor (CSF1R) blockade, targeting C‐X‐C motif chemokine ligand 12/C‐X‐C chemokine receptor type 4 (CXCL12/CXCR4) and agonistic CD40 mAb.

Also, in this issue of the *Annals of Gastroenterological Surgery*, Hazama et al beautifully summarize the development of specific immunotherapeutic strategies against gastrointestinal (GI) cancers, such as adoptive T‐cell transfer, peptide vaccines, or dendritic cell vaccines. They describe the strategies to resolve the immunosuppressive conditions surrounding the tumor microenvironment by using combination immunotherapy of ICI with peptide vaccines, novel immune adjuvants, COX‐2 inhibitors, and anti‐vascular endothelial growth factor receptors (anti‐EGFR) antibodies. Also, they proposed that novel next‐generation chimeric antigen receptor modified T cells (CAR‐T) therapy with expression of interleukin (IL)‐7 and chemokine (C‐C motif) ligand 19 (CCL19) was potentially promising for solid tumors including GI‐tract malignancy, as IL‐7 and CCL9 can attract dendritic cells (DC) and T cells into the tumor microenvironment.

It is generally accepted that the tumor microenvironment can be classified as having two distinct characteristics: one is the immunogenic tumor microenvironment (hot tumor), and another is the non‐immunogenic tumor microenvironment (cold tumor). Hot tumor consists of a significant amount of T‐cell infiltration with upregulation of programmed death ligand 1 (PD‐L1) expression on the tumor, whereas cold tumor has a faint infiltration of these cells. There is growing evidence that hot tumor tends to respond well to ICI, whereas cold tumor shows less response to ICI. Therefore, as for the cold tumor, combination immunotherapy of ICI with cancer vaccine, molecular target agents, or chemoradiation has a potential to induce cytotoxic T lymphocyte (CTL) infiltration and upregulation of PD‐L1 on tumors, leading to a conversion of cold tumor to hot tumor; thereafter, patients will be followed by ICI.

Taken together, so far, anti‐PD1 mAb is a key drug for immunotherapy for gastroenterological malignance. We can select responder patients for ICI based on biomarker profile using genome sequencing techniques and, finally, combination immunotherapy of ICI with other strategies may be an ideal or reasonable way to treat gastroenterological malignancy. Furthermore, there is a recent report on the development of personalized cancer immunotherapy in which the authors set up a process comprising comprehensive identification of individual mutations, computational prediction of neo‐epitopes, and design and manufacturing of a vaccine unique for each patient.^4^ We are facing new era of cancer immunotherapy.

## DISCLOSURE

Funding: Koji Kono was supported by a research grant from Taiho Pharmaceutical Company and Ono Pharmaceutical Company.

Conflict of Interest: Koji Kono received lecture fees from Ono Pharmaceutical Company.
